# What do clinicians edit in ambient AI-drafted clinical documentation? A qualitative content analysis

**DOI:** 10.1093/jamia/ocag073

**Published:** 2026-05-12

**Authors:** Yawen Guo, Di Hu, Ziqi Yang, Seungjun Kim, Brian Tran, Jamie Lee, Sitha Vallabhaneni, Rachael Zehrung, Sairam Sutari, Steven Tam, Emilie Chow, Danielle Perret, Deepti Pandita, Kai Zheng

**Affiliations:** Department of Informatics, University of California, Irvine, Irvine, CA 92617, United States; Department of Informatics, University of California, Irvine, Irvine, CA 92617, United States; Department of Informatics, University of California, Irvine, Irvine, CA 92617, United States; Department of Informatics, University of California, Irvine, Irvine, CA 92617, United States; Department of Informatics, University of California, Irvine, Irvine, CA 92617, United States; Department of Informatics, University of California, Irvine, Irvine, CA 92617, United States; Department of Informatics, University of California, Irvine, Irvine, CA 92617, United States; Department of Informatics, University of California, Irvine, Irvine, CA 92617, United States; Institute for Clinical and Translational Science, University of California, Irvine, Irvine, CA 92697, United States; Department of Medicine, University of California, Irvine, Irvine, CA 92617, United States; Department of Medicine, University of California, Irvine, Irvine, CA 92617, United States; Department of Physical Medicine & Rehabilitation, University of California, Irvine, Irvine, CA 92697, United States; Department of Medicine, University of California, Irvine, Irvine, CA 92617, United States; Department of Informatics, University of California, Irvine, Irvine, CA 92617, United States

**Keywords:** ambient listening, artificial intelligence, electronic health records[E05.318.308.940.968.625.500], qualitative coding, thematic analysis

## Abstract

**Objective:**

Ambient artificial intelligence (AI) documentation is increasingly used to draft clinical notes from patient-provider conversations, but how clinicians revise and finalize these drafts is not well understood. This qualitative content analysis study characterizes real-world edits to AI-generated drafts and identifies opportunities for improvement of AI design and the implementation process.

**Materials and Methods:**

Eight coders analyzed clinical documentation generated by ambient AI from 200 clinical encounters. We developed an inductive coding framework with 11 codes across 3 categories: clinical content, terminology, and language style. Interrater reliability was assessed using Cohen’s kappa. We then applied thematic analysis to synthesize patterns across the coded edits.

**Results:**

The most frequently edited content pertained to clinical facts including orders (eg, procedures, lab tests) (40.0%), symptoms (30.3%), medication prescriptions (27.3%), and diagnosis descriptions (25.9%). In comparison, edits related to terminology use (11.6%) and language style (7.2%) were less frequent. The results of our thematic analysis show that most edits can be categorized into one of the following 5 types: to revise factual discrepancies, to add medical specialty-specific details, to express diagnostic certainties, to convert patient expressions into objective assessments recorded in medical terms, and to reorganize or condense content.

**Conclusion and Discussion:**

Clinicians routinely revise ambient AI drafts to modify factual details and clinical specificity. Future work on AI development and clinical implementation should emphasize specialty customization and support personalized documentation practices, alongside clinician education that promotes robust and consistent review routines to ensure documentation quality.

## Introduction

Ambient artificial intelligence (AI) systems are increasingly integrated into clinical workflows to support documentation by generating note drafts.^1^^,^^2^ Early studies suggest they may reduce documentation burden, improve efficiency, and enhance note quality.^3^^,^^4^ However, despite growing adoption, the real-world reliability and clinical appropriateness of AI-drafted documentation remain underexplored.

Prior evaluations have largely emphasized clinician and patient perspectives through surveys and interviews, focusing on usability, trust, and time savings rather than the content of the generated text.^5–9^ A few studies compared AI-generated notes with reference text using automated performance and text-similarity metrics including the F1 score, overlap-based measures such as Recall-Oriented Understudy for Gisting Evaluation (ROUGE) and embedding-based scores. However, these approaches typically produce aggregate, note-level outcomes without explicating what content was changed.^10–12^ Expert-based note quality assessments, such as structured reviewer ratings and instruments like the Physician Documentation Quality Instrument, provide an overall quality assessment but are often conducted on simulated or curated data, offering limited insight into the specific edits clinicians make or the reasons behind them.^13^

Limited work has compared real-world ambient AI drafts and clinician-finalized notes in the electronic health record (EHR) systems to directly characterize editing behavior. Understanding these edits is essential because they show where AI drafts fall short and can reveal systematic gaps with implications for clinical safety, billing, and communication.^14^ They can also inform model improvement and implementation support to better align ambient AI with clinical documentation practice.

To address this gap, we conducted a content analysis of 314 ambient AI-generated note sections from 200 paired ambient AI drafts and clinician-finalized versions, including 1804 word-level edit operations (insertions, deletions, and replacements) across 33 specialties and 73 clinicians in an ambient AI pilot program. Using qualitative coding and thematic analysis, we examined the types of textual changes clinicians made, the content areas most frequently modified, and the broader themes that describe how clinicians refine AI-generated drafts into finalized documentation. This study offers a fine-grained empirical characterization of real-world clinician editing in ambient AI documentation, with the goal of informing more reliable and more workflow-aligned documentation support systems.

## Method

Informed by a post-positivist orientation, this study used a qualitative descriptive design with inductive content analysis and thematic analysis to characterize clinician edits to AI-generated clinical note drafts at the content level. This approach aligned with the study’s focus on describing patterned variation in observable text changes rather than developing theory or inferring clinicians’ underlying motivations. The research was conducted as part of a quality improvement initiative at the University of California, Irvine Medical Center (UCI Health), involving clinicians across ambulatory primary and specialty care clinics using 2 commercially available ambient AI systems that were integrated into the Epic electronic health record (EHR) via mobile and web interfaces. Vendors are referred to as Vendor A and Vendor B to preserve contractual and institutional confidentiality. By the end of the study period, Vendor A had been implemented for approximately 10 months and Vendor B for approximately 9 months. The study protocol was approved by the University of California, Irvine Institutional Review Board (IRB #7123). All data processing occurred within HIPAA-compliant secure computing environments, including UCI Health’s Protected Virtual Computing Environment (PVCE) and Amazon Web Services (AWS) infrastructure.

### Data source and sampling strategy

Ambient AI drafts clinical content by sections, covering history of present illness (HPI), assessment and plan (A&P), physical exam, and results, which clinicians can selectively insert into EHR notes. The overall corpus of this study consisted of all AI-generated draft note sections and their corresponding clinician-finalized versions recorded between September 26, 2024, and August 5, 2025, from the University of California, Irvine Medical Center. To construct an annotation dataset for content analysis that reflects the full corpus, we drew a stratified sample of 200 notes, each containing one or more AI-drafted note sections with clinician edits. Because this was an initial qualitative study intended to characterize clinician editing behavior across the full ambient AI deployment, we sampled broadly across all 33 specialties rather than focusing on a single specialty. This design allowed us to identify recurring cross-specialty edit patterns and common areas for tool improvement. Sampling quotas were aligned with the overall corpus distribution, ensuring the inclusion of at least 2 notes per specialty or one in cases where only a single note was available. Quotas were also designed to achieve a balanced representation from both vendor systems in the annotated sample.

### Edit detection using the myers diff algorithm

**Figure 1.**
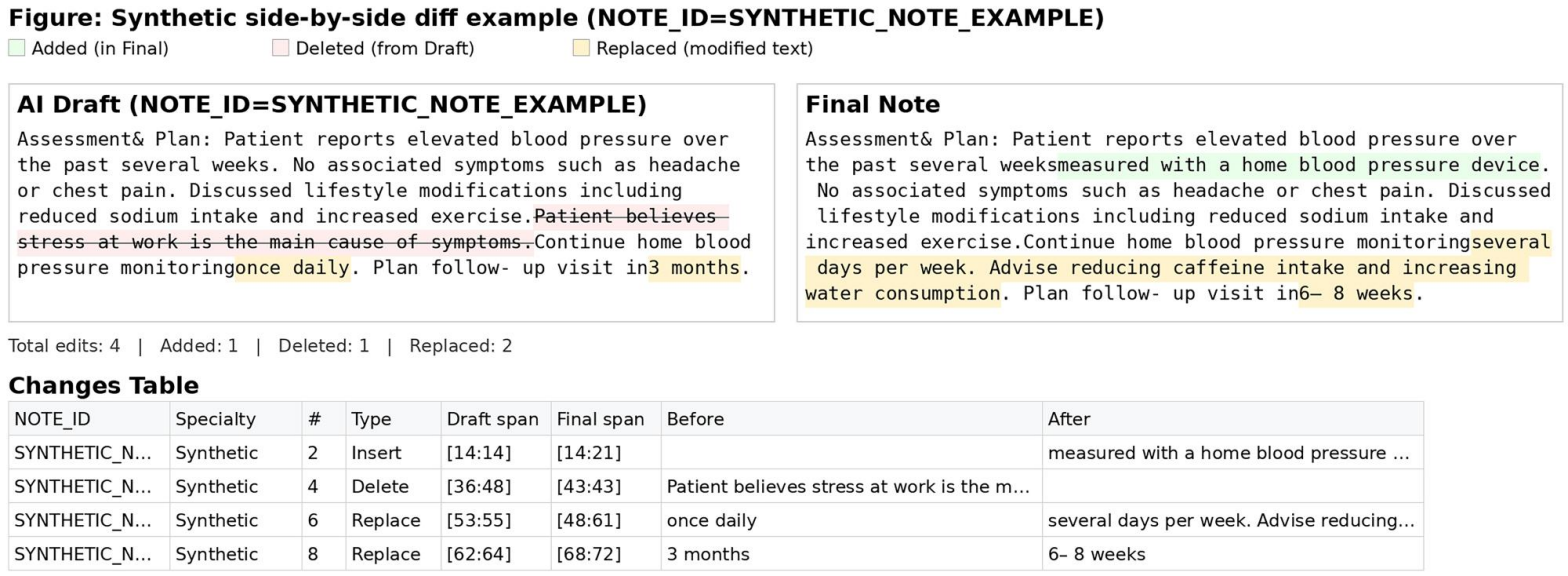
Synthetic example of side-by-side visualization of clinician edits to an AI-generated clinical note.

We detected editing activity in the sample by quantifying insertions, deletions, and replacements between each ambient AI draft and the clinician-finalized note section. For each draft–final pair, we applied a word-level Myers diff algorithm to align tokens and identify edit operations, recording the span and sequence of each change.^15^ We also generated side-by-side HTML views with highlighted edits, allowing for direct comparison of the AI output against clinician revisions. Figure 1 provides a synthetic example for illustration only and does not contain patient ata.

### Sentence-level content analysis

We defined an edit unit as the smallest contiguous set of sentences that fully captured each diff-detected edit span, including adjacent or overlapping edits, to preserve clinical context and meaning. To align draft and final text at the sentence level, we used a dynamic approach that allowed flexible mappings, including one-to-one, one-to-many (split), many-to-one (merge), and many-to-many alignments. Each edit unit was labeled by its alignment pattern and quantified by the number of insertions, deletions, and replacements it contained, capturing both within-sentence phrasing changes and larger structural revisions.

### Coding framework and procedure

An inductive coding framework was developed de novo to characterize the types of clinician edits.^16^ Two researchers (YG, DH) independently reviewed a subset of edit units to develop an initial codebook. Saturation in edit patterns was reached after independent review of sentence-level edit units from approximately 70 notes, after which no substantively new edit categories were identified. We then coded a stratified sample of 200 notes to ensure broad representation across both vendor systems and a wide range of clinical specialties. The codebook was then iteratively refined through consensus meetings and multiple rounds of coding with 8 coders, including 7 PhD students (YG, DH, ZY, SK, JL, SV, and RZ) and 1 MD-PhD student (BT), all with experiences in health informatics research and qualitative analysis. The full coding team then conducted an initial calibration round in which all 8 coders applied the draft codebook to the same set of edit units. We finalized the codebook after iterative calibration rounds, adjudication of disagreements, and refinement of code definitions until recurring coding ambiguities had stabilized; interrater reliability was then summarized using Cohen’s κ across major code categories.^17^ The final refined framework consisted of 3 top-level code categories capturing distinct categories of editing behavior (entity-level clinical content changes, terminology and wording adjustments, or language and style modifications), along with 11 subcodes representing more granular phenomena (Table 1).^18^^,^^19^

**Table 1.** Qualitative coding schema.

Primary code category	Code	Example(s)	Description
Entity-level: Clinical Content	Medication (E–Med)	Before: “Keppra one pump BID”; After: “Keppra 500 mg BID”	Edits that add, remove, or modify medication information, including medication name, dose, frequency, route, formulation, timing, or adherence details.
	Symptom (E–Sym)	Before: “Cramping”; After: “Cramping at night”	Edits that add, remove, or modify symptom mentions, including presence/absence, severity, location, timing, duration, triggers, or associated features.
	Diagnosis (E–Dx)	Before: “epilepsy”; After: “refractory focal epilepsy”	Edits that add, remove, or modify diagnoses/conditions or diagnostic labels, including specificity (type/subtype), qualifiers, or diagnostic framing.
	Procedure/test/lab order (E–Test)	Before: “EEG not done”; After: *(deleted)*	Edits to tests or procedures and related orders, including labs and imaging, whether performed in-clinic or externally, such as changes to test type, status, referrals, timing/location, or results.
	Demographics/ID (E–ID)	Before: “[WRONG-SPELLED NAME]”; After: “[CORRECTED NAME]”	Edits that add, remove, or modify patient identifiers or demographic details (eg, age, name, pronouns, PHI placeholders)
	Social context (E–Soc)	Before: *(none)*; After: “lives with spouse”	Edits that add, remove, or modify social history/context (eg, tobacco/alcohol/substance use, living situation, education, occupation, support system, marital status, habits/behaviors)^20^
	Past medical history (E-Hx)	Before: (none); After: “Previously saw nephrologist at [LOCATION]”	Edits that add, remove, or modify past medical history, including comorbidities, prior events, prior specialty care, surgeries, and relevant historical conditions.
Terminology and Wording	Lay–jargon substitution (T–Lay)	Before: “pain”; After: “myalgia”	Edits that substitute clinical jargon with lay language (or vice versa) while preserving the underlying meaning.
	Abbreviation Expansion/Contraction (T–Abbr)	Before: “right arm”; After: “R arm”	Edits that expand, introduce, or standardize abbreviations and acronyms.
Language and Style	Empathy/rapport/politeness (S–Emp)	Before: *(none)*; After: “It was a pleasure to see Ms. [NAME]”	Edits that change interpersonal tone (rapport, empathy, politeness, patient-centered phrasing) without changing core clinical content.
	Certainty/hedging (S–Cert)	Before: “due to”; After: “likely due to”	Edits that increase or decrease expressed certainty, probability, attribution, or diagnostic commitment (eg, hedging, qualifiers, speculative language).

Using the finalized codebook, coders applied labels to each sentence-level edit unit. We treated each code as a binary label, present or absent, for each sentence-level edit unit, allowing for multiple codes per unit. Across 8 coders, we computed Cohen’s kappa for each code for each annotator pair and macro-averaged kappas across codes with equal weight; overall reliability was the mean of the 28 pairwise macro-averaged kappa values. Because edit units often reflected specialty-specific documentation practices and patient-specific clinical context, we anticipated partial overlap at code boundaries and therefore used iterative calibration and consensus adjudication to ensure a stable codebook prior to thematic synthesis. All coding was conducted in structured spreadsheets using the finalized codebook. To maintain independent coding, each coder worked in a separate spreadsheet. For reference when needed, coders could consult color-coded HTML diff visualizations of the paired draft and final text to view each edit unit alongside the exact textual changes within the full note section.

### Thematic analysis

After all edit units were coded using the finalized codebook, we conducted a thematic synthesis to identify higher-level patterns in clinician revision behavior. Vendor identities were masked during codebook development and edit-unit coding to reduce bias and support consistent application of the codebook. After coding was complete, vendors were specified for a vendor-stratified descriptive summary of code distributions and to assess whether themes were represented in both vendor groups. Two lead researchers (YG and DH) independently reviewed code distributions, representative edit units, and analytic memos to examine how frequently co-occurring codes reflected shared underlying revision behavioral patterns. Codes were iteratively grouped based on conceptual similarity, co-occurrence patterns, and recurring forms of observable revision in the paired draft-final notes. Through this process, we aggregated code-level edits into 5 higher-level themes that capture recurring clinician revision patterns in AI-drafted notes. Themes were refined through iterative team discussion and disagreements in theme boundaries were resolved through consensus. The resulting themes represent interpretive syntheses of multiple code types and are intended to characterize how clinicians systematically revise AI-drafted documentation.

## Results

### Dataset characteristics

A total of 314 note section pairs from 200 notes containing ambient AI drafted sections and finalized versions were included in the annotated sample, representing 73 clinicians, 200 patients, and 33 clinical specialties. Clinicians could generate more than 1 ambient AI section draft within the same note (for example, only A&P, or both HPI and A&P). We coded section-level draft–final pairs, grouped by note ID for tracking. The complete sampled notes for qualitative annotation included a history of present illness section (*n* = 149) and an assessment and plan section (*n* = 135), while fewer included physical examination (*n* = 18) or results (*n* = 12). Across all sampled draft–final section pairs, clinicians made 1804 token-level modifications, including 556 insertions, 393 deletions, and 855 replacements. The sample covered 33 specialties; family practice contributed the most notes (*n* = 54), followed by rheumatology (*n* = 22) and internal medicine (*n* = 20), with the remaining notes distributed across a broad set of lower-volume specialties (Table 2).

**Table 2.** Characteristics for paired ambient AI drafts and clinician-finalized notes.

Category	Subcategory	Overall	Vendor A	Vendor B
Overall (sample-level)	Study period	2024-09-26 to 2025-08-05		
	# of notes	200	109	91
	# of clinicians	73	35	38
	# of patients	200	109	91
AI-drafted Note Section Pairs	# of History of Present Illness (HPI) pairs	149	73	76
	# of Assessment & Plan (A&P) pairs	135	87	48
	# of Physical Exam pairs	18	11	7
	# of Results pairs	12	5	7
Token-level editing activity	# of Insertions	556	311	245
	# of Deletions	393	198	195
	# of Replacements	855	466	389
Specialty Distribution (*n* = # of note)	Family Medicine	54	39	15
	Rheumatology	22	20	2
	Internal Medicine	20	17	3
	Orthopaedic Surgery	15	1	14
	Geriatric Medicine	15	0	15
	Others*	74	32	42

* Other specialties (Overall *n* = 74; Vendor A *n* = 32; Vendor B *n* = 42) included: Hand Surgery (A = 1, B = 9); Neurology (A = 0, B = 7); Nurse Practitioner (A = 4, B = 2); Gastroenterology (A = 3, B = 0); Ob/Gyn (A = 0, B = 3); Physician Assistant (A = 0, B = 3); Cardiology (A = 2, B = 0); Colon and Rectal Surgery (A = 0, B = 2); Dermatology (A = 2, B = 0); Emergency Medicine (A = 0, B = 2); Endocrinology (A = 2, B = 0); Epilepsy (A = 0, B = 2); General Surgery (A = 0, B = 2); Head and Neck Surgery (A = 0, B = 2); Hematology/Oncology (A = 0, B = 2); Hospitalist (A = 2, B = 0); Infectious Diseases (A = 2, B = 0); Integrative Medicine (A = 2, B = 0); Neurosurgery (A = 2, B = 0); Ophthalmology (A = 2, B = 0); Optometry (A = 2, B = 0); Otolaryngology (A = 2, B = 0); Physical Medicine and Rehab (A = 2, B = 0); Psychiatry (A = 0, B = 2); Urology (A = 1, B = 1); Vascular Neurology (A = 0, B = 2); Orthopedics (A = 0, B = 1); Vascular Surgery (A = 1, B = 0).

### Distribution of qualitative edit codes

We coded 713 edit units extracted from 314 AI draft–final note section pairs. Vendor A contributed 373 edit units and Vendor B contributed 340 edit units. After an initial training phase and 2 rounds of independent coding with consensus discussions, interrater reliability across 8 coders reached a moderate level of agreement (overall Cohen’s κ = 0.568), reflecting the heterogeneity and complexity of coding clinical edits;^21^ disagreements were resolved through adjudication and consensus discussions. Because a single edit unit could receive multiple codes, results are reported as the frequency of edit units containing each code; percentages therefore do not sum to 100 percent. Table 3 summarizes the distribution of edit codes overall and by vendor.

**Table 3.** Distribution of qualitative edit codes overall and by vendor.

Code	Vendor A, *n* (%)*	Vendor B, *n* (%)*	Total, *n* (%)*
Procedure/test/lab order (E-Test)	160 (42.9%)	125 (36.8%)	285 (40.0%)
Symptom (E-Sym)	121 (32.4%)	95 (27.9%)	216 (30.3%)
Medication (E-Med)	120 (32.2%)	75 (22.1%)	195 (27.3%)
Diagnosis (E-Dx)	111 (29.8%)	74 (21.8%)	185 (25.9%)
Past medical history (E-Hx)	73 (19.6%)	62 (18.2%)	135 (18.9%)
Social context (E-Soc)	60 (16.1%)	67 (19.7%)	127 (17.8%)
Demographics/ID (E-ID)	37 (9.9%)	26 (7.6%)	63 (8.8%)
Lay–jargon substitution (T-Lay)	25 (6.7%)	26 (7.6%)	51 (7.2%)
Abbreviation expansion/contraction (T-Abbr)	22 (5.9%)	10 (2.9%)	32 (4.5%)
Certainty/hedging (S-Cert)	17 (4.6%)	12 (3.5%)	29 (4.1%)
Empathy/rapport/politeness (S-Emp)	11 (2.9%)	11 (3.2%)	22 (3.1%)

* Percentages are calculated using the total number of coded edit units within each subgroup as the denominator (Vendor A: *N* = 373; Vendor B: *N* = 340; Overall: *N* = 713). Because an edit unit can receive multiple codes, counts may sum to >100%.

The most common edit types involved clinical content. Procedures, tests, and lab orders edits (E–Test) appeared in 285 edit units (40.0%). A common pattern was clarifying order-specific metadata, including the test type, the intended location or service, and the timing or status of the order when that detail affected interpretability. This was followed by symptom changes (E–Sym) in 216 (30.3%). For symptom edits, clinicians commonly refined the symptom description to improve clinical specificity. This included adjusting the symptom label, and specifying severity or pattern when the draft was vague. Clinicians also removed symptom descriptions that were not part of the patient’s current presentation or that appeared to be carried over from prior context without supporting evidence from the encounter. Medication modifications (E–Med) appeared in 195 (27.3%), and edits most often made the regimen actionable by adding or correcting dose, frequency, route, or administration details. Clinicians also standardized medication nomenclature, such as correcting the drug name, aligning the term with common clinical usage, or clarifying which formulation was intended. A smaller but recurring pattern was medication reconciliation, where clinicians removed a medication that was not relevant to the current plan or clarified whether the patient was currently taking it. Diagnosis refinements (E–Dx) appeared in 185 (25.9%). Clinicians frequently revised the diagnostic framing by narrowing a broad label to a more specific condition, aligning the diagnosis term with the symptom description, or clarifying whether the diagnosis was established versus under evaluation. Edits involving past medical history (18.9%) and social context information (17.8%) were also frequent, whereas terminology and wording edits such as jargon versus lay (7.2%) and abbreviation changes (4.5%) and language and style edits such as certainty or hedging (4.1%) and empathy and rapport (3.1%) were less common.

### Thematic synthesis of clinician edits

Editing activity varied by note section, with the highest concentration in narrative sections such as HPI and Assessment and Plan and fewer edits in Physical Exam and Results. Vendor-stratified summaries showed broadly similar code distributions, and all 5 themes were observed in notes from both vendors. Given the qualitative aim and descriptive nature of vendor stratification, we present themes pooled across vendors for interpretability. Representative before/after examples are shown in Supplementary Table S1.

#### Theme 1- Edits that revise factual discrepancies and specific clinical details in AI drafts

These edits revised misspelled names and pronouns, and time-related information in the draft notes. They also modified event descriptions by updating prior medical history details, such as changing a hospitalization to an emergency department visit, and by revising anatomic and severity information (eg, laterality, joint location, stenosis severity). Clinicians further edited medication and test/procedure details, including drug names and dosing frequency, sedation type, and imaging modality. These edits indicate frequent clinician revision of detail-level information, including medical history, demographics, and treatments.

#### Theme 2- Edits that refine generic drafts into specialty-appropriate documentation

Clinicians added specialty-specific clinical details and terminology that were absent or less developed in the original drafts, such as symptom onset and trajectory, medication regimen specifics (dose, schedule, and whether use was as needed), and test or treatment context that shaped the plan. In neurology/epilepsy, edits added temporal progression and neuroanatomical localization, expanding brief summaries into more differentiated problem-oriented assessments and documenting symptom course, differentiate neurologic problems, and document medication intervals or titration. For example, a neurology progress note was expanded by adding detailed trauma history, symptom characterization across neurologic problems, and rewriting the assessment into a specialized, multi-layered plan. In procedure-oriented specialties such as hand surgery and orthopaedics, edits replaced general descriptions with structured, more specific findings and procedure- or imaging-specific terminology, including digit-level exam metrics or dated radiology findings. A hand surgery note was revised by replacing general symptom and exam language with digit-specific, quantified findings (eg, finger-level localization, monofilament scores) and by adding clear procedural intent, such as proceeding with right index and ring trigger finger release. In cardiology, clinicians expanded symptom documentation beyond brief general phrasing by specifying exertional context and exercise tolerance, linking dyspnea, chest symptoms, or palpitations to what the patient can do and under what conditions, so the note reflected cardiovascular-style functional assessment rather than a generic symptom checklist.

#### Theme 3- Edits that revise the certainty level of diagnostic and causal statements

Across notes, clinicians often replaced definitive causal or diagnostic wording with more qualified language, such as changing “due to” to “likely due to” and revising “no retinal or optic nerve disease” to “no obvious retinal or optic nerve disease.” They also revised wording so that conditions were described as under evaluation rather than already established, for example, by reframing plans aiming to “definitively rule out or confirm AFib” as monitoring in the setting of “no confirmed diagnosis of AFib.” Similarly, diagnostic labels were revised alongside test information documented in the note, such as shifting a stated dermatomyositis diagnosis to a suspected drug eruption after a negative myositis panel.

#### Theme 4- Edits that replace conversational phrasing with standardized chart language and terminology

Clinicians frequently edited transcript-like language into more standardized chart phrasing, including removing or reframing patient-attributed causal explanations and speculative risk phrasing, and by replacing them with more concise clinician-facing statements. For example, subjective clauses such as “which she believes are related to her thyroid condition” or “she recalls an instance when…” were removed or rewritten as verifiable clinical information, such as the current levothyroxine dose or the patient’s expressed concern about thyroid level fluctuations. More broadly, conversational and vague phrasing was rewritten into conventional chart language and medical terminology, such as changing “she’s doing okay” to “patient reports stable condition” and replacing lay descriptions like “a disintegrating disc” with “a bulging disc.”

Edits also standardized abbreviations and shorthand, for example, using “MCV” and inserting routine clinical abbreviations such as “EGD,” “abnl,” and “CIN II.” Clinicians modified the drafts by naming the exact device, procedure, or structure, such as revising “partial amputation” to “ray amputation,” and specifying the ECU tendon. Medication references were similarly clarified by replacing generic wording with the specific drug name, such as “used the medication” to “used Qsymia” and “Lexapro” to “escitalopram,” with accompanying refinements to dosing or decision language.

#### Theme 5- Edits that reorganize, relocate, and condense AI drafts

Clinicians frequently restructure AI-generated drafts through reorganization, relocation, and condensation of content. Dense narrative was often converted into structured formats, for example, with long paragraphs rewritten as bullet lists, problem narratives turned into numbered plans, and free text reorganized under problem-oriented headings. Clinicians also relocated information to more different labeled sections, such as moving social or surgical history into labeled past history fields and separating imaging descriptions into a dedicated radiology-style section with dated, objective findings. Edits also included consolidation of duplicative or boilerplate text was consolidated, and removal of content that appeared misaligned with the clinical context, including passages on specialty-irrelevant medication management.

## Discussion

Clinicians substantially modify AI-generated drafts, and those edits follow repeated patterns that align with the 5 themes in our qualitative analysis, which point to specific opportunities for improvement in ambient AI documentation tools and their implementation.

Clinicians frequently revise apparent factual discrepancies and detail-level information in AI drafts, especially in discrete details that can change clinical meaning, such as names, medication dosages, and test or procedure attributes. Some of these revisions may reflect underlying inaccuracies or hallucinated content in the AI draft; however, this study did not independently verify the clinical correctness of the original draft or of every clinician revision. Even when the overall narrative in AI drafts appears broadly plausible, these discrepancies are difficult for downstream readers to detect and can propagate if copied forward. If left undetected and carried into the finalized note, they may threaten patient safety by distorting information used in subsequent clinical decision-making. These patterns suggest that improving factual reliability of ambient AI tools should be a top priority, paired with workflow features that help clinicians quickly verify the fields that are most likely to need review.^22^ At present, most ambient AI tools do not directly access the patient’s chart, so certain discrepancies, such as names or other identifiers, cannot be automatically cross-checked even when the correct information already exists in the EHR. If chart integration becomes feasible, some of these discrepancies may be reduced upstream. In the meantime, for institutions integrating ambient AI into routine practice, training for clinicians should go beyond a generic requirement to “review the draft” and instead teach a consistent verification routine that prioritizes the most high-risk details such as medication names and dosages, test and procedure attributes, patient identifiers, and the certainty level of diagnostic or treatment-related statements.^23^^,^^24^

The recurring addition of specialty-specific detail suggests that a single, generic note structure and style is often insufficient for specialty documentation needs. An actionable response is to support specialty-tuned drafting behavior through templates and prompts that better support inclusion of details that matter for a given discipline, rather than broadly relying on the subjective–objective–assessment–plan (SOAP) structured format.^25–27^ For institutions, this implies that ambient AI adoption should be paired with specialty-level configuration and evaluation aligned with each specialty’s documentation norms and clinical priorities.^28^

The recurring revision of certainty level in diagnostic and causal language suggests that some AI-generated phrasing may be more definitive than clinicians preferred in the finalized note. Beyond clinical risk, this pattern may raise medicolegal concerns if AI-generated phrasing systematically nudges notes toward stronger diagnostic commitments than the encounter supports.^29^ In our data, clinicians frequently replaced more definitive wording with more qualified phrasing. Actionably, AI developers can in response design models that default to appropriately qualified language and conventional diagnostic framing, and that avoid stating conclusions more strongly than the note supports.^30^ At the practice level, this theme points to a clear focus for governance and education, reinforcing a consistent review habit around diagnostic uncertainty and helping reduce the risk that system outputs encourage overly definitive documentation when diagnoses are not yet established.

Clinicians often replaced transcript-like phrasing with more standardized chart language and terminology. This mismatch may be amplified by how the system structures conversational content into the Subjective section (SOAP).^24^ In our data, clinicians frequently condensed and reorganized that section, suggesting potential misalignment between how the draft is assembled and how clinicians typically write and review notes. Ambient AI developers can strengthen attribution-aware writing that keeps a clear boundary between patient reports and clinician assessment without adding interpretation that the note does not support. Terminology normalization can also help increase precision without causing note bloat.^31–34^ For clinicians, this theme is a reminder that review is not only error correction; it is also ensuring that the finalized note is written in a form that supports efficient downstream reading and interpretation.

Recurring patterns of note restructuring and condensing suggest that draft organization may be an important factor in how clinicians revise ambient notes for use in practice. For example, ambient capture can include social history and social determinants of health (SDOH) information that is clinically relevant because it can shape access to care, home and community support, and adherence. However, in the context of certain encounters, some of these elements may be secondary to the immediate diagnostic and clinical decision making, particularly when captured with high granularity (eg, transportation needs narratives).^35^ In our data, clinicians often shortened, relocated, or removed these details from the draft. One possible interpretation is that these edits reflect real-world tradeoffs between completeness and note manageability. As notes get longer, they become harder to review efficiently, and important information can be harder to find.^36^^,^^37^ An actionable implication is to prioritize tool behaviors that produce more structured drafts, route content into predictable sections, and give clinicians simple ways to control verbosity without losing clinically important context. At the organizational level, this supports setting expectations for note structure and routinely monitoring note length and usability as part of ambient AI governance.

This study has several limitations. Our qualitative analysis drew on the AI draft–final note section pairs from 200 notes, and some specialties were represented by only a small number of cases, so we may not fully capture specialty-specific nuances. We did not quantify temporal trends in this annotated sample, and clinician editing behavior may change over time as users gain experience with ambient AI. Future large-scale computational analyses of edit types could help evaluate these potential learning-curve effects more directly. The dataset also comes from a single health system and a single deployment context, which may limit how well these patterns generalize to other institutions, care settings, or ambient AI products. Vendor-stratified summaries are descriptive, and the study was not designed or powered for formal between-vendor comparisons. The modest qualitative sample and broad thematic categories might limit sensitivity to subtle vendor-specific differences. Interrater agreement was moderate (overall Cohen’s κ = 0.568). This is consistent with a multi-coder, multi-label codebook applied to heterogeneous notes spanning diverse specialties and patient contexts, where code boundaries can overlap for clinically adjacent edits. We mitigated this through iterative calibration, adjudication, and consensus discussions, and we generated themes based on convergent patterns across codes and examples rather than any single coder’s label.^38^ We only analyzed text changes without the source audio, user interface cues, or clinicians’ stated intent. Therefore, we cannot determine why a given edit was made or whether it reflects individual style, local documentation norms, or risk tolerance. Finally, we did not assess the clinical correctness of the finalized notes or downstream impacts such as care outcomes, time burden, or corresponding change of billing codes.

Taken together, our findings translate routine clinician edits into concrete actionable insights for both tool improvement and real-world implementation. The patterns we observed suggest that ambient AI should be evaluated and tuned for specialty needs and clinician-to-clinician variation. Future work should use targeted specialty-level samples and more granular specialty-specific codebooks to capture domain-specific documentation needs, such as treatment regimen details in oncology or procedure-specific planning language in surgical care. Additionally, we plan to conduct targeted semi-structured interviews to clarify the rationales and workflow factors behind common edits and to better understand clinicians’ reasoning behind these patterns. Future work should also investigate whether specific edit patterns are associated with continuity of care. For example, edits that clarify the problem list or tighten the assessment and plan may make notes more usable for downstream readers. Studies should also examine operational performance, including turnaround time, downstream review effort or follow-up work, and whether term-level changes affect billing or coding outcomes. Finally, future work should evaluate safety-critical signals, especially apparent factual discrepancies in AI drafts and revisions to diagnostic or causal certainty language when diagnoses remain uncertain.

## Conclusion

Our qualitative content analysis of clinician edits to AI drafts provides a comprehensive view of what clinicians changed and where improvement is needed. Clinicians frequently revised apparent factual discrepancies and specific clinical details, added specialty-specific information and terminology to otherwise generic drafts, and replaced more definitive diagnostic or causal wording with more qualified language. They also rewrote conversational, patient-attributed narrative into professional documentation and reorganized drafts into clearer, more structured notes. Ambient AI tools should be designed around how clinicians document in different specialties and should avoid wording that goes beyond the evidence in the encounter. Health systems can use recurring edit patterns to identify target implementation support and training that improve day-to-day documentation efficiency.

## Supplementary Material

ocag073_Supplementary_Data

## Data Availability

The underlying dataset contains protected health information (PHI) and is not publicly available.
