# Supplementary material for: What do clinicians edit in ambient AI-drafted clinical documentation? A qualitative content analysis
Source: J Am Med Inform Assoc. 2026 May 12;33(8):1457–65. doi: 10.1093/jamia/ocag073 (PMC13386006; doi:10.1093/jamia/ocag073)
Supplement: ocag073_Supplementary_Data [file ocag073_supplementary_data.docx]

Supplementary Table S1. Representative examples of clinician edits to ambient AI–drafted notes by five qualitative themes.

| ***Theme 1- Edits that revise factual discrepancies and specific clinical details in AI drafts.*** | | |
| --- | --- | --- |
| **Edit type** | **AI draft text** ^a^ | **Clinician-final text** ^a^ |
| Demographic correction | “Seen by [PROVIDER_NAME_A].” | “Seen by [PROVIDER_NAME_B].” |
| Demographic correction | “The patient is [AGE_A] years old.” | “The patient is [AGE_B] years old.” |
| Temporal correction | “...in March” | “...in next March” |
| Tense correction | “The patient has been taking...” | “The patient had been taking...” |
| Pronoun correction | “...he...” | “...she...” |
| Clinical event type correction | “a recent hospitalization” | “a recent ER visit on 12/15/24” |
| Medication detail correction | “has been using fluconazole 200 mg weekly” | “has been using fluconazole 200 mg a few times per week” |
| Medication name correction | “Zepal” | “albuterol” |
| Laterality correction | “Cerumen impaction… more on the left” | “Cerumen impaction… more on the right” |
| Anatomic site correction | “MCP joint” | “DIP joint” |
| Procedure/test label correction | “colonoscopy” | “sigmoidoscopy” |
| Test detail correction | “MRI of brain” | “MRA of head and neck” |
| Device name correction | “a cardio device” | “a Kardia device” |
| Followup detail correction | “return in 6 week” | “return in 2–3 Month” |
| ***Theme 2- Edits that refine generic drafts into specialty-appropriate documentation*** | | |
| **Edit type** | **AI draft text** | **Clinician-final text** |
| Diagnosis specificity | “headache” | “possibly migraine with aura” |
| Diagnosis specificity | “hypoglycemia and hyperglycemia” | “hyperglycemia and rare hypoglycemia” |
| Diagnosis specificity | “Psoriatic Arthritis…” | “Rheumatoid arthritis, double seropositive… high titer anti-CCP” |
| Symptom specificity | “noise sensitivity” | “light and sound sensitivity” |
| Symptom specificity | “pain in back and neck” | “neck pain without acute changes” |
| Symptom specificity | “Achilles” | “Calf pain” |
| Temporal course specification | “headaches” | “persistent daily headaches since November 2024” |
| Medication regimen specification | “Vitamin B2” | “Vitamin B2 400 mg per day” |
| Medication regimen specification | “Prescribe meloxicam once daily…” | “Restart meloxicam 15 mg daily with dinner.” |
| ***Theme 3- Edits that revise the certainty level of diagnostic and causal statements.*** | | |
| **Edit type** | **AI draft text** | **Clinician-final text** |
| Certainty calibrated to evidence | “due to” | “likely due to” |
| Certainty calibrated to evidence | “No retinal or optic nerve disease.” | “No obvious retinal or optic nerve disease.” |
| Certainty calibrated to evidence | “to definitively rule out or confirm AFib” | “as there is currently no confirmed diagnosis of AFib.” |
| ***Theme 4- Edits that replace conversational phrasing with standardized chart language and terminology.*** | | |
| **Edit type** | **AI draft text** | **Clinician-final text** |
| Clinical register normalization | “She’s doing okay” | “Patient reports stable condition” |
| Medical terminology standardization | “a disintegrating disc” | “a bulging disc” |
| Clinician framing / documentation precision | “He is on HIV medications.” | “He is compliant with HIV medications.” |
| Abbreviation standardization | “mean corpuscular volume” | “MCV” |
| Technical specificity increase | “potential removal of the stones” | “potential removal of the dropped gall stones in hepatorenal fossa” |
| Procedure terminology standardization | “partial amputation” | “ray amputation” |
| Generic to specific entity | “used the medication” | “used Qsymia” |
| Medication name normalization | “Lexapro” | “escitalopram” |
| Patient narrative reframed into clinician documentation | Subjective self-attributions (“which she believes are related…”, “she recalls…”) | Reframed into objective clinician documentation |
| ***Theme 5- Edits that reorganize, relocate, and condense AI drafts.*** *^b^* | | |
| **Edit type** | **AI draft structure** | **Clinician-final structure** |
| Format conversion | Long paragraph | Bullet list (e.g., “• Continue healthy diet…”) |
| Format conversion | Problem paragraph (prose) | Numbered list (1–7) |
| Format conversion | Prose narrative | Structured “#condition:” headings |
| Section relocation | Social + surgical history embedded mid-paragraph | Moved under labeled “past medical history” |
| Relevance trimming | Large chunks of past history | Deleted and/or reorganized; “psychiatric history” section added/structured |

*^a^ Examples are de-identified; bracketed tokens denote masked identifiers, and excerpts are paraphrased where needed to protect privacy.*

*^b^ For Theme 5, edits span longer passages and section organization; we therefore report structure-level transformations rather than verbatim text.*
